# Identification of New Candidate Genes and Chemicals Related to Esophageal Cancer Using a Hybrid Interaction Network of Chemicals and Proteins

**DOI:** 10.1371/journal.pone.0129474

**Published:** 2015-06-09

**Authors:** Yu-Fei Gao, Fei Yuan, Junbao Liu, Li-Peng Li, Yi-Chun He, Ru-Jian Gao, Yu-Dong Cai, Yang Jiang

**Affiliations:** 1 Department of Surgery, China-Japan Union Hospital of Jilin University, Changchun 130033, People’s Republic of China; 2 Institute of Health Sciences, Shanghai Institutes for Biological Sciences, Chinese Academy of Sciences, Shanghai 200031, People’s Republic of China; 3 College of Life Science, Shanghai University, Shanghai 200444, People’s Republic of China; University of Georgia, UNITED STATES

## Abstract

Cancer is a serious disease responsible for many deaths every year in both developed and developing countries. One reason is that the mechanisms underlying most types of cancer are still mysterious, creating a great block for the design of effective treatments. In this study, we attempted to clarify the mechanism underlying esophageal cancer by searching for novel genes and chemicals. To this end, we constructed a hybrid network containing both proteins and chemicals, and generalized an existing computational method previously used to identify disease genes to identify new candidate genes and chemicals simultaneously. Based on jackknife test, our generalized method outperforms or at least performs at the same level as those obtained by a widely used method - the Random Walk with Restart (RWR). The analysis results of the final obtained genes and chemicals demonstrated that they highly shared gene ontology (GO) terms and KEGG pathways with direct and indirect associations with esophageal cancer. In addition, we also discussed the likelihood of selected candidate genes and chemicals being novel genes and chemicals related to esophageal cancer.

## Introduction

Esophageal cancer refers to tumors located in the region from the esophagus between the throat and stomach and has become the eighth most common cancer in recent years. Symptoms of esophageal cancer include difficulty in swallowing and vomiting blood. Based on data from SEER 18 2004–2010, the five-year survival rate of this cancer was approximately 17.5% [1].

There are two main subtypes of esophageal cancer, squamous-cell carcinoma and adenocarcinoma [2,3], which can be classified based on their location and ancestral precancerous cells. Smoking and alcohol likely contribute to both subtypes. Nitrosamines and nutritional deficiencies are among the main causes of squamous-cell carcinoma, while gastroesophageal reflux disease (GERD) and obesity are linked to adenocarcinoma. Different clinical therapies have been used to treat esophageal cancer, including surgery, chemotherapy and radiotherapy [4].

Mutations in multiple genes including p53, FasL, and EGFR have been reported to be associated with the onset and development of esophageal cancer [5–9]. Changes in gene expression levels have also been linked to the cancer; for example, induced expression of MAL was found in esophageal cancer [10] and inactivation of RUNX3 may correlate with esophageal carcinogenesis [11]. Recent studies have also examined the effect of miRNAs [12].

Despite progress, our understanding of esophageal cancer is still limited and the identification of affected genes is still far from complete. Furthermore, most current studies concentrate on annotated genes and therefore overlook other useful information, such as small biological molecules. Here, we attempted to simultaneously identify novel genes and chemicals related to esophageal cancer.

Computer science has been greatly developed over the past few decades, making it possible to utilize large scale datasets. Many biological problems involving large scale data, such as protein-protein interactions, chemical-chemical interactions, and complicated biological networks, have been partly or completely tackled by designing effective computational methods [13–22]. Encouraged by the success of these studies, we investigated the mechanisms contributing to esophageal cancer by analyzing the related data using computers. Recently, a group of computational methods based on the shortest path algorithm was proposed to study various diseases by searching novel disease genes in protein-protein interaction networks [23–25]. However, these methods only considered disease genes.

Other popular graph methods include Guilt-by-association [26] and Random Walk with Restart (RWR) [27–32]. The assumption of Guilt-by-association was that a gene and its nearest neighbors are more likely to share similar roles and functions on a network and thereafter it tends to be a disease gene if most of its neighbors are disease genes [33]. RWR assumed that the disease signal can be propagated from known disease genes to unknown ones through the network. It simulates a random walker who starts from the known disease genes and then randomly walks to their neighbors on the network. After many steps, the probabilities of all genes on the network to be visited by the walker will become stable and such probabilities can be used to represent the possibility of a gene being a disease gene [29].

In the paper, we generalized the shortest path method to simultaneously study genes and chemicals by constructing a hybrid interaction network containing both proteins and chemicals. Based on known genes and chemicals related to esophageal cancer, we searched for new candidate genes and chemicals by applying a shortest path algorithm to the hybrid interaction network. Next, a permutation test was adopted to control false discoveries. We compared our generalized shortest path method with RWR on a comprehensive dataset of esophageal cancer. Based on jackknife test, our shortest path method outperforms or at least performs at the same level as the RWR. Enrichment analysis of the final obtained genes indicated that they shared some gene ontology (GO) terms and KEGG pathways related to esophageal cancer. In addition, we also discussed the likelihood of some of the relationships between the new candidate genes and chemicals and esophageal cancer. We hope that this contribution provides new insights for understanding esophageal cancer and benefits the clinical field of this disease.

## Materials and Methods

### Genes and chemicals related to esophageal cancer

Current known human genes related to esophageal cancer were collected from three datasets: (I) Uniprot (http://www.uniprot.org/, release 2014_4) [34]; (II) the TSGene Database (Tumor Suppressor Gene Database, http://bioinfo.mc.vanderbilt.edu/TSGene/cancer_type.cgi) [35]; and (III) the NCI (National Cancer Institute, https://gforge.nci.nih.gov, released 2009.6) database [36]. In detail, a total of 144 reviewed genes related to esophageal cancer were obtained from Uniprot by inputting "human”, “esophagus cancer” and “reviewed" as the keywords; 155 genes were obtained from the TSGene Database after the Entrez IDs were converted to the official symbols; and four genes were obtained from NCI by selecting genes that were recorded as esophagus cancer/esophagus carcinoma-related genes. After combining the aforementioned genes and removing redundancy, we finally obtained 156 unique genes related to esophageal cancer. These genes are listed in S1 File.

Chemicals related to esophageal cancer were retrieved from CTD (Comparative Toxicogenomics Database, http://ctdbase.org/) [37], a widely used database for chemical and disease associations. The associations were manually summarized based on 110,142 articles (http://ctdbase.org/about/dataStatus.go, accessed in August 2014). We downloaded 59 chemicals with a known mechanism in esophagus carcinoma, therapeutic chemicals for esophagus carcinoma and marker chemicals of esophagus carcinoma. However, we only considered the 30 chemicals occurring in the constructed hybrid interaction network (see Section 2.2). The pubchem IDs of these chemicals are also provided in S1 File.

### Construction of the hybrid interaction network

To construct a hybrid interaction network, we downloaded information on protein-protein interactions from STRING (Search Tool for the Retrieval of Interacting Genes/Proteins, version 9.1, http://www.string-db.org/) [38] and information on chemical-chemical interactions and protein-chemical interactions from STITCH (Search tool for interactions of chemicals, version 4.0, http://stitch.embl.de/) [39]. The interactions provided by STRING and STITCH include both known and predicted interactions. Each interaction was labeled with a score to measure the likelihood of the interaction’s occurrence. For formulation, let us denote the score of the interaction between proteins *p*_1_ and *p*_2_ by *I*_*pp*_(*p*_1_, *p*_2_), the score of the interaction between chemicals *c*_1_ and *c*_2_ by *I*_*cc*_(*c*_1_, *c*_2_), and the score of the interaction between protein *p* and chemical *c* by *I*_*pc*_ (*p*, *c*). To reduce the search space, we only considered chemicals with records in KEGG (Kyoto Encyclopedia of Genes and Genomes) [40].

The constructed hybrid interaction network placed the retrieved proteins and chemicals as nodes. Two nodes were linked by an edge if and only if the corresponding proteins or chemicals could comprise an interaction. Furthermore, to indicate the strength of the interaction each edge *e* was assigned a weight by

$$w(e)=\{\begin{array}{ll} 1000-I_{pp}(p_{1},p_{2}) & \mathrm{If}\;\text{the endpoints of}\;e\;\text{represented proteins}\;p_{1}\;\text{and}\;p_{2}\\ 1000-I_{pc}(p,c) & \mathrm{If}\;\text{the endpoints of}\;e\;\text{represented protein}\;p\;\text{and chemical}\;c\\ 1000-I_{cc}(c_{1},c_{2}) & \mathrm{If}\;\text{the endpoints of}\;e\;\text{represented chemicals}\;c_{1}\;\text{and}\;c_{2} \end{array}$$ (1)

Since the maximum value of the interaction score is 999, we used 1000 minus the interaction score as the weight of the corresponding edge. This allowed the shortest path algorithm to execute on this network in order to find new candidate genes and chemicals.

### Method to find new candidate genes and chemicals

Based on known genes and chemicals related to esophageal cancer mentioned in Section 2.1, we searched all shortest paths in the hybrid interaction network that connect any pair of known genes or chemicals. The genes and chemicals (excluding the known genes and chemicals) that were identified in any of the shortest paths as inner nodes were labeled candidate genes and chemicals. To distinguish these candidate genes and chemicals for further selection, we counted the number of shortest paths containing certain candidate genes or chemicals as inner nodes. This value was termed the betweenness in this study. This selection procedure contained a permutation test that constructed 1,000 randomly selected gene and chemical sets. The number of genes and chemicals in each of the randomly selected sets were the same as that of the set consisting of known genes and chemicals. For each randomly selected set, search all shortest paths in the network which connect any two genes or chemicals in the set. Based on these paths, calculate the betweenness of each candidate gene and chemical. Therefore, for each candidate gene or chemical, there are one betweenness on the known gene and chemical set and 1,000 betweenness on 1,000 randomly selected sets. The permutation P-value was computed as “the number of randomly selected sets for which the betweenness was greater than that of the known gene and chemical set”/1000. Finally, we set a P-value of 0.05 as the threshold for exclusion of candidate genes and chemicals. The detailed procedure of the method and its principle can be found in previous studies [23–25].

### Comparing to the Random Walk with Restart (RWR) method

Random Walk with Restart (RWR) method is widely applied to identify disease genes [27–32]. Let’s use *P*_*t*_ to denote the state probabilities at time *t*; *P*_*0*_ to denote the initial state probabilities, a column vector assigning 1/m to each of the *m* known esophageal cancer gene and chemical nodes and 0 to other nodes on the hybrid network; and A′ to denote the column-wise normalized adjacency matrix which represented the structure of the hybrid network. The restart probability, *r*, was set to be 0.7 as suggested by a previous study [29]. The state probabilities *P*_*t*+1_ at time *t* + 1 can be calculated as following

$$P_{t+1}=\left(1-r\right)A^{\prime }P_{t}+r$$ (2)

When the difference between *t* + 1 and *t* was smaller than 1e-6 as suggested by a previous study [29], the random walker stopped the walk and the state probabilities of all nodes on the network became stable. The nodes with higher probabilities were more likely to be disease-related. To evaluate the significance of the possible disease nodes, we performed permutations of known disease nodes 1,000 times and the permutation P-value was “the number of randomly selected sets with which the probability was greater than that of the known gene and chemical set”/1000. The genes and chemicals with P-value smaller than 0.05 were considered as candidate disease genes and chemicals.

We applied jackknife test to compare the performances of RWR with our method. Each time, we excluded one known disease node in turn as the positive test sample and used all other disease nodes (the training samples) to train the model. The trained model will assign a P-value to all test samples including all the negative samples and the positive test sample. According to the P-values, we predict the test samples to be disease or non-disease related. If we assumed all negative samples were not related to the disease and only the excluded positive sample was related to the disease, then the F1-measure can be calculated as following

$$\text{F}1=\frac{2\text{r}\times \text{p}}{\text{r}+\text{p}}$$ (3)

$$\text{r}=\frac{\text{tp}}{\text{tp}+\text{fn}}$$ (4)

$$\text{p}=\frac{\text{tp}}{\text{tp}+\text{fp}}$$ (5)

where r, p, tp, fp and fn stand for recall, precision, true positive, false positive, false negative, respectively.

After all known disease nodes were tested, we calculated the average F1-measure of the jackknife test to score the performance of the method.

## Results and Discussion

### Candidate genes and chemicals

By searching the shortest paths connecting any pair of known genes or chemicals related to esophageal cancer, 463 new genes and 90 new chemicals were extracted. These genes and chemicals and their betweenness values are listed in S1 Table. Next, these candidate genes and chemicals were filtered using a permutation test. The permutation P-values of these genes and chemicals are also provided in S1 Table. Finally, we obtained 164 genes and 24 chemicals with permutation P-values smaller than 0.05. These genes and chemicals were deemed to be significant for esophageal cancer and termed significant candidate genes and chemicals. Readers can refer to S2 Table for detailed information of these genes and chemicals.

**Fig 1:**
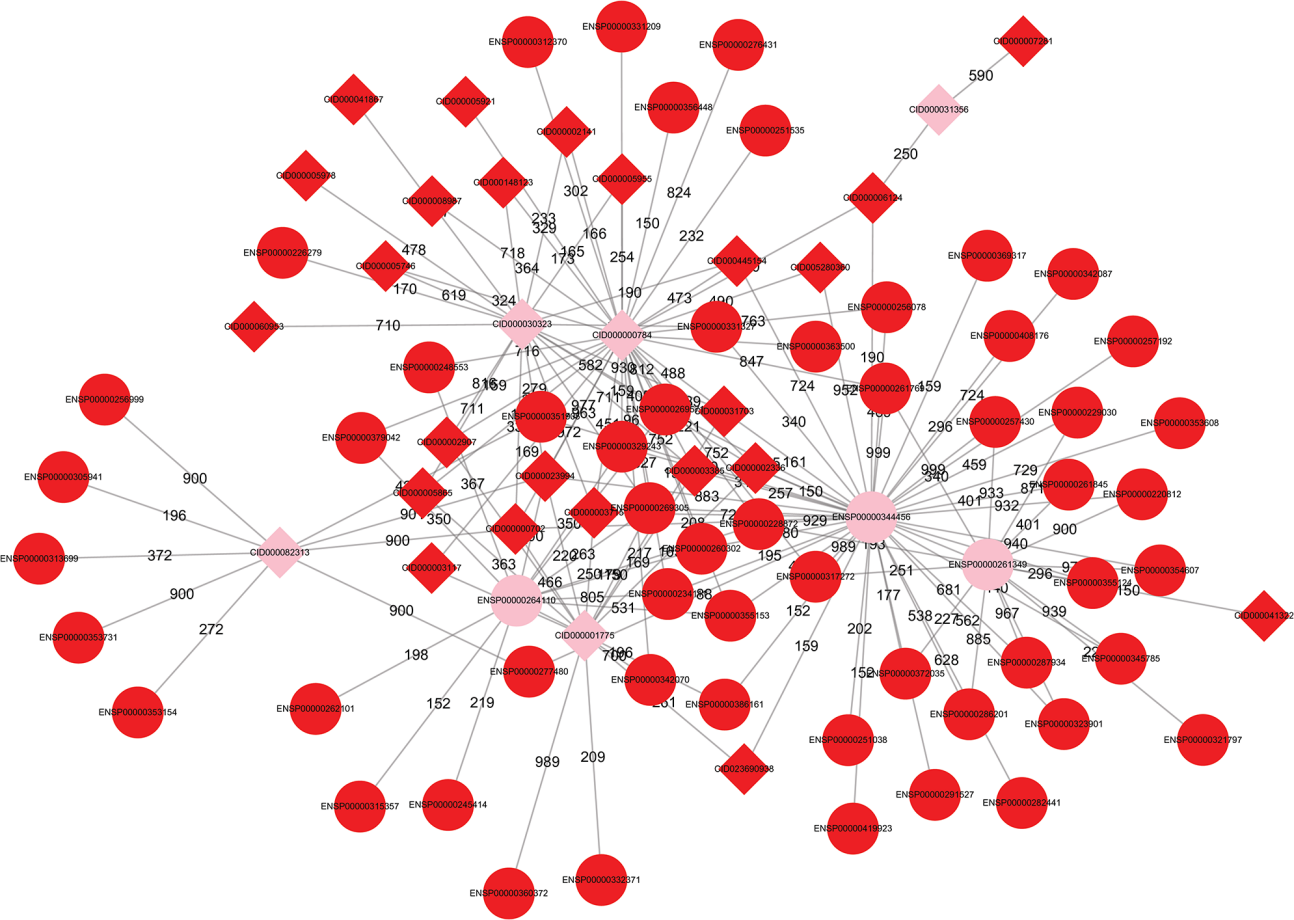
The network of highly possible novel esophageal cancer related genes and chemicals and known genes and chemicals related to esophageal cancer. The pink nodes were highly possible novel esophageal cancer-related genes and chemicals. The red nodes were known genes and chemicals related to esophageal cancer. The round nodes were genes and the diamond nodes were chemicals. The edge labels were the confidence scores of the interaction.

We listed some novel genes and chemicals related to esophageal cancer based on the permutation P-value, betweenness and literature surveys in **Table 1**. These genes and chemicals receive high confidence scores for esophageal cancer on the network as shown in **Fig 1**.

**Table 1:** Selected significant candidate genes and chemicals deemed to be closely related to esophageal cancer.

Protein or chemical ID	Name	Betweenness	Permutation P-value
ENSP00000344456	CTNNB1	2294	<0.001
ENSP00000261349	LRP6	698	<0.001
ENSP00000264110	ATF2	428	<0.001
CID000000784	Hydrogen Peroxide	353	0.046
CID000031356	TRIS	177	0.001
CID000001775	Phenytoin	10	0.04
CID000030323	Daunomycin	2	0.046
CID000082313	N-Acetyl-d-glucosamine	177	0.002

### Analysis of significant candidate genes

In our study, 164 significant candidate genes were obtained and are listed in S2 Table. Next, we investigated the probability of some of them being novel genes related to esophageal cancer. Rows 2–4 of **Table 1**list the information concerning the discussed genes.

#### CTNNB1

The betweenness and permutation P-value of CTNNB1 (catenin beta1, Ensembl ID: ENSP00000344456) were 2,294 and 0, respectively (see row 2 of **Table 1**). CTNNB1 is part of the adherens junction (AJ) and plays an essential role in the Wnt pathway. The phosphorylation of ERK1/2 can be decreased by down-regulation of β-catenin expression, thereby arresting cell cycle progression [41]. Abnormal β-catenin expression has been detected, with reduced expression inhibiting cell proliferation of esophageal carcinoma cells [42,43]. The detailed mechanism is not clear and needs further research. MUC1 (mucin 1) is a transmembrane mucin. Its aberrant expression in esophageal squamous cell carcinoma is associated with invasion or metastasis [44]. It has been speculated that MUC1 induces metastasis in esophageal cancer, suggesting that MUC1 may be developed as a diagnostic biomarker and drug therapeutic target.

#### LRP6

The betweenness and permutation P-value of LRP6 (low density lipoprotein receptor-related protein 6, Ensembl ID: ENSP00000261349) were 698 and 0, respectively (see row 3 of **Table 1**). LRP6 is a transmembrane receptor for the Wnt signaling pathway and plays a key role in cell proliferation, differentiation and migration in various types of cancers, including esophageal cancer. In the Het-1A esophageal epithelial cell line, expression of LRP6 was increased by MSE (mainstream cigarette smoke extract) stimulation in a manner that could be inhibited by pSSBP2 (single-stranded DNA-binding protein2) transfection [45]. These results indicate that LRP6 is an essential gene associated with esophageal carcinogenesis linked to cigarette smoking. Axin1 is also a negative regulator of the Wnt pathway; it interacts with APC, Gsk-3β and β-catenin to promote Gsk-3β-mediated phosphorylation and β-catenin degradation. LRP6 is phosphorylated by Gsk-3β under stimulation by Wnt, implying that LRP6 has an indirect relationship with Axin1 [46,47]. Mutations in Axin1 are associated with many types of cancer, with a 12% deletion frequency in medulloblastoma and detrimental mutations in HCC (hepatocellular carcinomas) [48,49]. Rare mutations of Axin1 were detected in esophageal SCC, while reduced expression of Axin was observed in oesophageal squamous cell carcinoma [50]. Despite controversial research concerning Axin1, we considered the idea that LRP6 and Axin1 are novel key elements in esophageal cancer through their effects on the Wnt pathway [50,51].

#### ATF2

The betweenness and permutation P-value of ATF2 (activating transcription factor 2, Ensembl ID: ENSP00000264110) were 428 and 0, respectively (see row 4 of **Table 1**). ATF2 belongs to the leucine zipper family of DNA binding proteins and performs multiple biological functions. The ATF2 protein can be phosphorylated by stress-activated MAPKs-JNK or p38 in response to extracellular stresses, such as UV light, hypoxia and DNA damage [52–54]. Furthermore, ATF2 can be phosphorylated and activated by the RalGDS-Src-P38 pathway and Ras-MEK-ERK [55]. ATF2 functions differently in different cancers, acting as an oncogene in melanoma and a tumor suppressor in breast cancer [56,57]. The expression of JNK, Ras and their pathways are significantly changed in esophageal cancer, but no evidence linking ATF2 to these signaling cascades in esophageal cancer has been reported [58,59]. Our results reveal the ATF2 was emphasized with a significant P-value, implying that the function of ATF2 is instrumental in esophageal cancer.

### Analysis of significant candidate chemicals

In this study, we also obtained 24 significant candidate chemicals for esophageal cancer (see S2 Table). The rest of this section discusses the likelihood of some of them being novel chemicals related to esophageal cancer. Information pertaining to the discussed chemicals is listed in rows 5–9 of **Table 1**.

#### Hydrogen Peroxide

The betweenness and permutation P-value of hydrogen peroxide (Pubchem ID: CID000000784) were 353 and 0.046, respectively (see row 5 of **Table 1**). Reactive oxygen species (ROS) are a type of free radical containing oxygen and include hydrogen peroxide (H_2_O_2_). ROS mediate cell growth and angiogenesis in cancer by regulating growth factors and transcription factors [60–64]. ROS are involved in tumor initiation and tumor progression of many cancer types, including melanoma, breast carcinoma and fibrosarcoma [65–68]. A variety of tumor cell lines and tumor tissues, such as lung cancer and breast cancer, produce H_2_O_2_ [69–71]. H_2_O_2_ production is activated by PDGF and EGF, two growth factors expressed by vascular smooth muscle cells and the A431 cell lines, respectively [60,61]. H_2_O_2_ acts as a direct or indirect signal molecule in regulation of PKC, MAPK, JAK, Ras, NF-kB, c-Myc and AP-1, which are closely correlated with carcinogenesis, including esophageal cancer [72]. Increased production of H_2_O_2_ may stimulate the activity of tyrosine phosphorylation, MAP kinase and DNA synthesis in a manner that can be reversed by H_2_O_2_ inhibitors [60,61]. H_2_O_2_ production stimulates the transcription of MMPs and the adhesion of cancer cells, which can be inhibited by catalase (CAT) [73]. Through analysis of the correlation of small molecules and target genes we identified MMP13, which may be stimulated by H_2_O_2_ and impact esophageal cancer cell adhesion. Furthermore, H_2_O_2_ may be a chemical carcinogenesis factor in metal-mediated DNA damage and genetic alteration in normal epithelial cells and cancer cells [74]. Cancer cell apoptosis can be induced by over-production of H_2_O_2_, which is induced directly or indirectly by widely used clinical drugs such as paclitaxel and arsenic trioxide [75–77]. p53 is a key element in DNA damage repair and cell apoptosis. We propose that p53 is an important factor that acts between the influence of H_2_O_2_ on normal cells and down-stream biological mechanisms and behaviors.

#### TRIS

The betweenness and permutation P-value of TRIS (Pubchem ID: CID000031356) were 177 and 0.001, respectively (see row 6 of **Table 1**). Tris (2,3-dibromopropyl) phosphate (TRIS) is a flame retardant used in synthetic fabrics, particularly polyesters, as well as cotton, wool and rayon [78]. There is evidence from bacterial systems that TRIS is a mutagenic factor [79,80] and there has been widespread publicity concerning its carcinogenicity in mice and rats [81]. Dietary experiments showed that TRIS can induce benign or malignant tumors of the forestomach and lung in female and male mice, the kidney in male mice and the liver in female mice [82]. Dermal exposure to TRIS can induce damage to the skin, forestomach, lung and oral cavity in female mice [83]. Dermal exposure to TRIS in rabbits revealed an analogous phenomenon [84]. Long-term exposure to TRIS may cause carcinomas. Although TRIS is considered as a carcinogen, the mechanism of the carcinogenic process is unclear. In addition to the tumor types discussed above, we propose that TRIS is a putative carcinogen for esophageal cancer, requiring future research to discover its mechanism and validate the clinical data.

#### Phenytoin

The betweenness and permutation P-value of phenytoin (Pubchem ID: CID000001775) were 10 and 0.04, respectively (see row 7 of **Table 1**). Phenytoin is a widely used antiepileptic drug that blocks voltage-gated Na^+^ channels (VGSC) (IC50~10μM) and K^+^ channels (HERG) at IC50>300 μM concentrations [85–87]. Voltage-gate channels also play essential roles in the development and potentiation of cancers through regulation of a broad range of cellular activities. Phenytoin can inhibit MDA-MB-231(a human breast cancer cell line) migration and invasion through control of Na^+^ channels [88]. In small cell lung cancer cells, endocytosis was suppressed by phenytoin [86,89]. Human ether-a-go-go related gene 1 (hERG1) channels are a type of K^+^ channel that regulates cellular secretion in various types of cells and exhibits dysfunction in many types of cancer [90–92]. In Barrett’s Esophagus, hERG1 channels are aberrantly expressed [93]. As discussed above, we propose phenytoin may be a putative therapeutic drug for esophageal cancer, but further investigation is required. The details of the mechanism are not clear, but through analysis of phenytoin and related genes we identified highlighted genes CYP2C19, CTSB and p53. Cytochrome P450, family 2, subfamily C, polypeptide 19 (CYP2C19) is a member of the cytochrome P450 superfamily of enzymes, which catalyze many biochemical reactions including drug metabolism. The CYP2C19-regulated drug metabolic system may be closely correlated with phenytoin treatment of cancers. Furthermore, Cathepsin B (CTSB), a lysosomal cysteine proteinase, was aberrantly altered in esophageal cancer. In Barrett’s esophagus and esophageal adenocarcinoma patients 8p23.1 (containing CTSB) is frequently amplified [94]. CTSB is also aberrantly expressed in epileptogenesis [95]. Thus, CTSB may be a novel putative phenytoin-regulated target.

#### Daunomycin

The betweenness and permutation P-value of daunomycin (Pubchem ID: CID000030323) were 2 and 0.046, respectively (see row 8 of **Table 1**). Daunomycin is an antibiotic obtained from Streptomyces peucetius that consists of a daunomycinone and an amino sugar (daunosamine). Daunomycin is used to treat some types of cancer, most commonly specific types of leukemia [96,97]. As an antitumor antibiotic, daunomycin intercalates DNA and presumably interferes with DNA-dependent RNA synthesis [98,99]. In esophageal cancer treatment, daunomycin may be an effective putative chemotherapy drug. In our study, we found that P53, ERBB2, CD38 and WT1 have putative relationships with daunomycin. P53, at the center of tumorigenesis, regulates down-stream genes involved in transcription, cell cycle progression and cell apoptosis. We speculated that daunomycin acts as an impact factor in the p53 center network directly or indirectly to inhibit tumor cell proliferation and induce cell apoptosis. CD38 is a novel multifunctional ectoenzyme that functions in cell adhesion and signal transduction. Our data indicate that CD38 and its network are also target factors impacted by daunomycin. In future studies, more focus needs to be given to the p53 and CD38 networks regulated by daunomycin.

#### N-Acetyl-d-glucosamine

The betweenness and permutation P-value of N-Acetyl-d-glucosamine (Pubchem ID: CID000082313) were 177 and 0.002, respectively (see row 9 of **Table 1**). N-Acetyl-d-glucosamine (N-Acetylglucosamine, or GlcNAc) is a monosaccharide derivative of glucose that participates in several biological systems. It is a key component of the bacterial cell wall and the extracellular matrix in animals [100]. In multicellular organisms, the O-linked attachment of GlcNAc regulates intracellular proteins at the posttranslational modification level that are involved in many cellular functions [101,102]. It was proposed that GlcNAc may affect branched N-glycans on cell surface proteins, thereby dysregulating the function of metabolic systems in diseases such as cancers [103]. FOLH1 was highlighted by our interaction analysis. FOLH1 (folate hydrolase (prostate-specific membrane antigen 1) is a type II transmembrane glycoprotein that is up-regulated in cancerous cells. Although there is no direct evidence that proves the interaction between FOLH1 and GlcNAc, GlcNAc is a novel modified factor for the FOLH1 protein. In future research, their relationship and the mechanism involved with the signaling pathway requires functional validation in animal models.

### Analysis of enriched KEGG pathways of significant candidate genes

**Fig 2:**
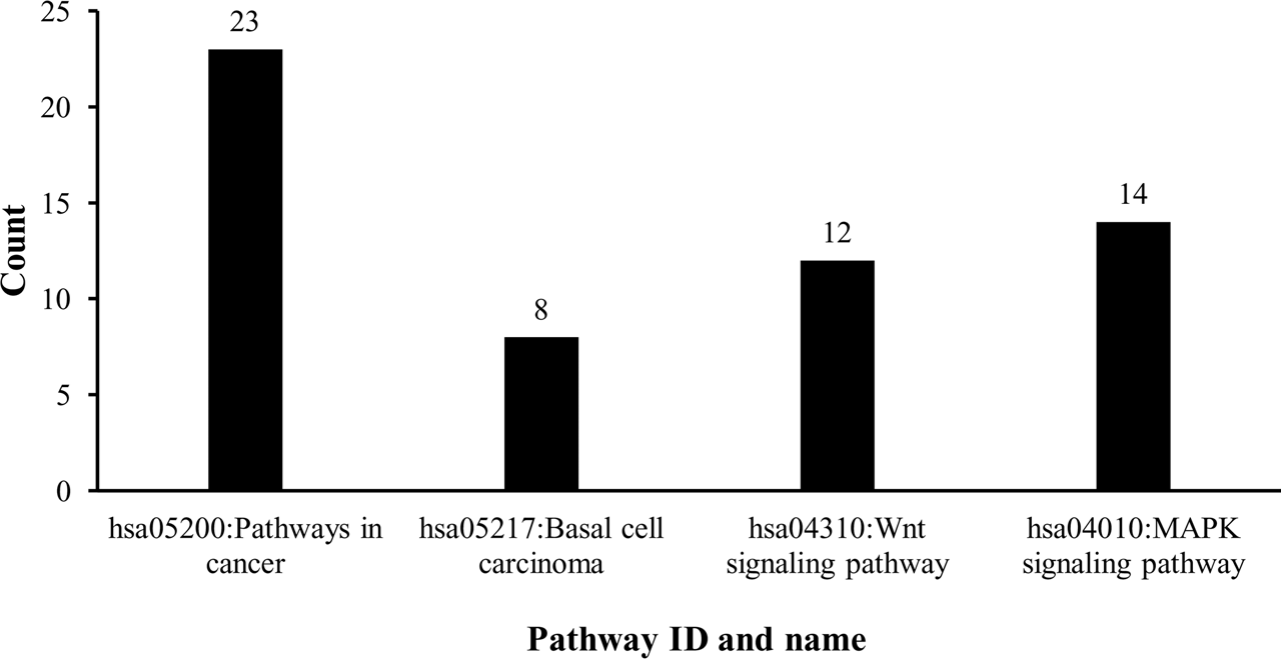
The four KEGG pathways that were highly shared by 164 significant candidate genes and highly associated with esophageal cancer. The X-axis indicates the pathway ID and name, while the Y-axis represents the number of genes that shared the pathway.

We obtained 164 significant candidate genes and 24 significant candidate chemicals potentially related to esophageal cancer pathogenesis. To analyze the relationship between them and esophageal cancer, we employed DAVID (Database for Annotation, Visualization and Integrated Discovery) [104], a functional annotation tool used to understand the biological meaning behind large lists of genes. DAVID provided enrichments for the 164 significant candidate genes based on KEGG pathways and GO terms, which are provided as S3 and S4 Tables, respectively. It can be seen from S3 Table that a total of 17 KEGG pathways were shared by the 164 significant candidate genes. Among the top five pathways with P-values less than 0.01, four were highly associated with esophageal cancer. **Fig 2**shows these pathways and the number of genes among the 164 significant candidate genes that shared each pathway (‘count’).

The most enriched pathway was hsa05200: pathways in cancer, with 23 significant candidate genes sharing this pathway (see **Fig 2**). The second was hsa05217: basal cell carcinoma, with 8 significant candidate genes (see **Fig 2**). This result indicates that a common mechanism is shared by esophageal cancer and other types of cancer. The third pathway was hsa04310: Wnt signaling pathway, with 12 significant candidate genes (see **Fig 2**). The Wnt pathway acts as a common element in regulation of stem cell renewal and maintenance of many systems. Disruption of this pathway is also associated with other cancers [105]. For example, Wnt1 is involved in embryonic development, which often calls for rapid cell division and migration. Dysregulation of these processes can cause abnormal cell growth and movement, which can lead to tumor development [106]. Inhibition of Wnt signaling may prove to be an effective method for inhibition of the uncontrolled renewal that drives cancers, including esophageal cancer. The fourth pathway was hsa04010: MAPK signaling pathway, with 14 significant candidate genes (see **Fig 2**). The mitogen-activated protein kinases (MAPKs) include extracellular signal-regulated kinase (ERK), p38, and c-Jun NH2-terminal kinase (JNK). Many cancer-associated mutations of components of the MAPK signaling pathways have been found in Ras and B-Raf, both of which participate in the ERK signaling pathway [107]. The ERK pathway can also induce expression of matrix metalloproteinases, promote degradation of extracellular matrix proteins and contribute to consequent tumor invasion [108].

Although these significantly enriched pathways have been previously reported to be related to esophageal cancer, our results may expand new avenues of research, resulting in exploration of new mechanisms of esophageal cancer pathogenesis.

### Analysis of enriched GO terms of significant candidate genes

**Fig 3:**
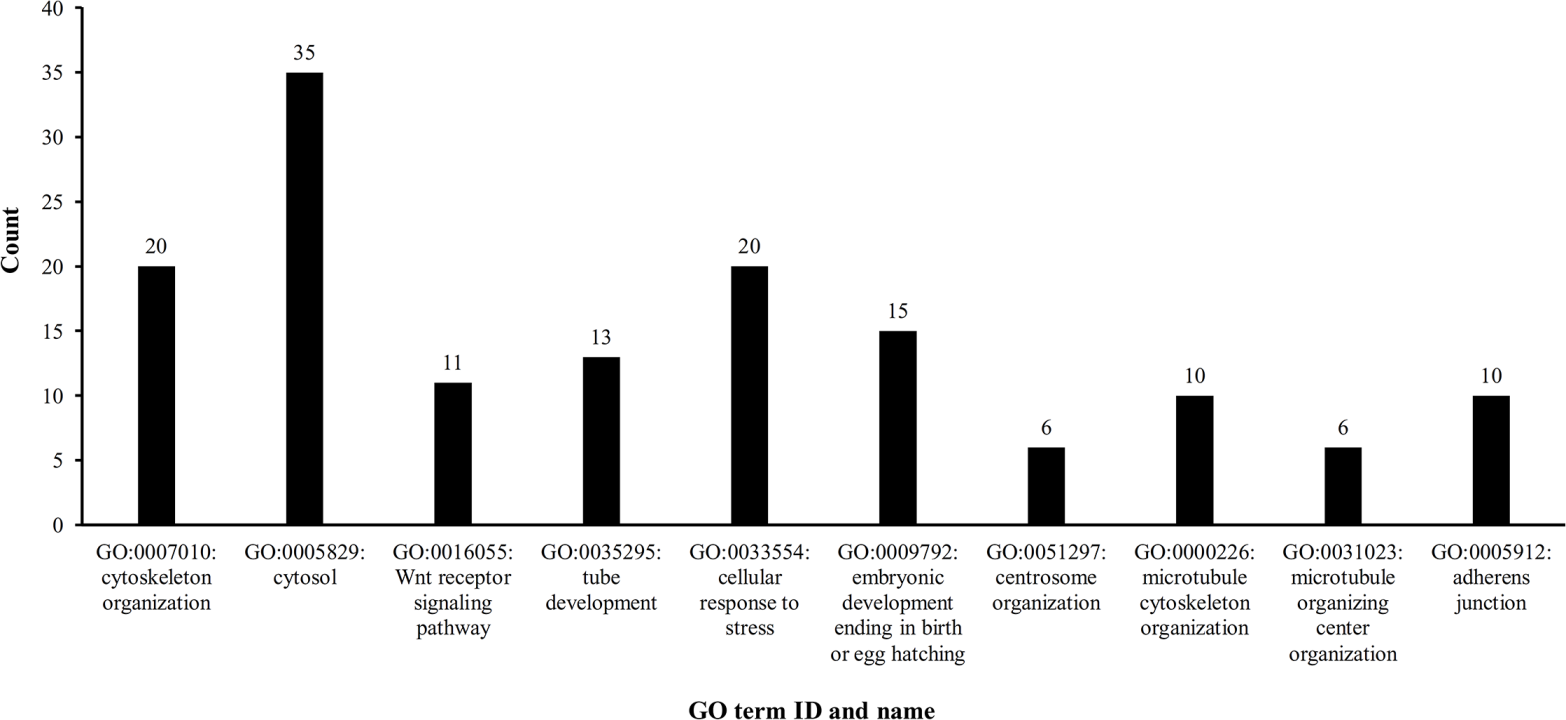
The top ten GO terms that were highly shared by 164 significant candidate genes. The X-axis indicates the GO term ID and name, while the Y-axis represents the number of genes that shared the pathway.

As shown in S4 Table, 473 GO terms were enriched by the 164 significant candidate genes. The top ten GO terms sorted by P-value were investigated. **Fig 3**shows these GO terms and the number of genes among the 164 significant candidate genes that shared the GO term (‘count’).

Among these GO terms, eight were biological process (BP) GO terms and two were cellular component (CC) GO terms. Seven GO terms were highly associated with cytoskeleton, microtubule and adherens junctions, including five BP terms: GO:0007010: cytoskeleton organization (‘count’ = 20, refer to **Fig 3**); GO:0035295: tube development (‘count’ = 13, refer to **Fig 3**); GO:0051297: centrosome organization (‘count’ = 6, refer to **Fig 3**); GO:0000226: microtubule cytoskeleton organization (‘count’ = 10, refer to **Fig 3**); and GO:0031023: microtubule organizing center organization (‘count’ = 6, refer to **Fig 3**). Additionally, two CC terms were significant: GO:0005829: cytosol (‘count’ = 35, refer to **Fig 3**) and GO:0005912: adherens junction (‘count’ = 10, refer to **Fig 3**). Importantly, esophageal cancer can spread to other tissues and organs. Common sites of spread include nearby lymph nodes, the liver, lungs and bone [109]. Actin and microtubule cytoskeletons are key players that underpin cellular processes, including defects in cellular morphogenesis, the acquisition of inappropriate migratory and invasive characteristics, and defects in mitosis [110].

Additionally, GO:0016055: Wnt receptor signaling pathway (‘count’ = 11, refer to **Fig 3**) is highly related to esophageal cancer, as discussed in Section 3.2. Several genes in the Wnt pathway, such as Wnt1, are also involved in embryonic development, which often calls for rapid cell division and migration [106]. Because of this association, GO:0009792: embryonic development ending in birth or egg hatching (‘count’ = 15, refer to **Fig 3**) is also included. GO:0033554: cellular response to stress (‘count’ = 20, refer to **Fig 3**) can reflect the circumstances of the esophageal mucosa. Esophageal cancer has two main sub-types. Smoking tobacco, drinking alcohol and drinking very hot drinks are among the most causes of the squamous variety, while smoking and acid reflux are among the most common causes of adenocarcinoma [111]. Chemicals in tobacco, alcohol, high temperature and gastric acid can all change the intracellular and extracellular environment of the esophageal mucosa, resulting in stress to mucosal cells.

### Comparing our method to RWR

First, we used jackknife test to evaluate the performance of our method and RWR on all known genes or chemicals related to esophageal cancer. The average F1-measure of RWR was 0.000402, lower than the F1-measure of our method which was 0.000431. This indicates that our method outperforms or at least performs at the same level as RWR.

Furthermore, we applied the RWR method to predict candidate genes related to esophageal cancer and compared the GO and KEGG enrichment results for three gene sets: known esophageal cancer related genes, shortest path predicted genes (*i*.*e*., candidate genes predicted by our method) and RWR predicted genes, using DAVID. The enrichment p value was transformed into enrichment score, *i*.*e*.,-log_10_(p value). The enrichment scores of all GO terms and KEGG pathways that were enriched in at least one gene set with p value smaller than 0.1 were listed in S5 Table.

**Fig 4:**
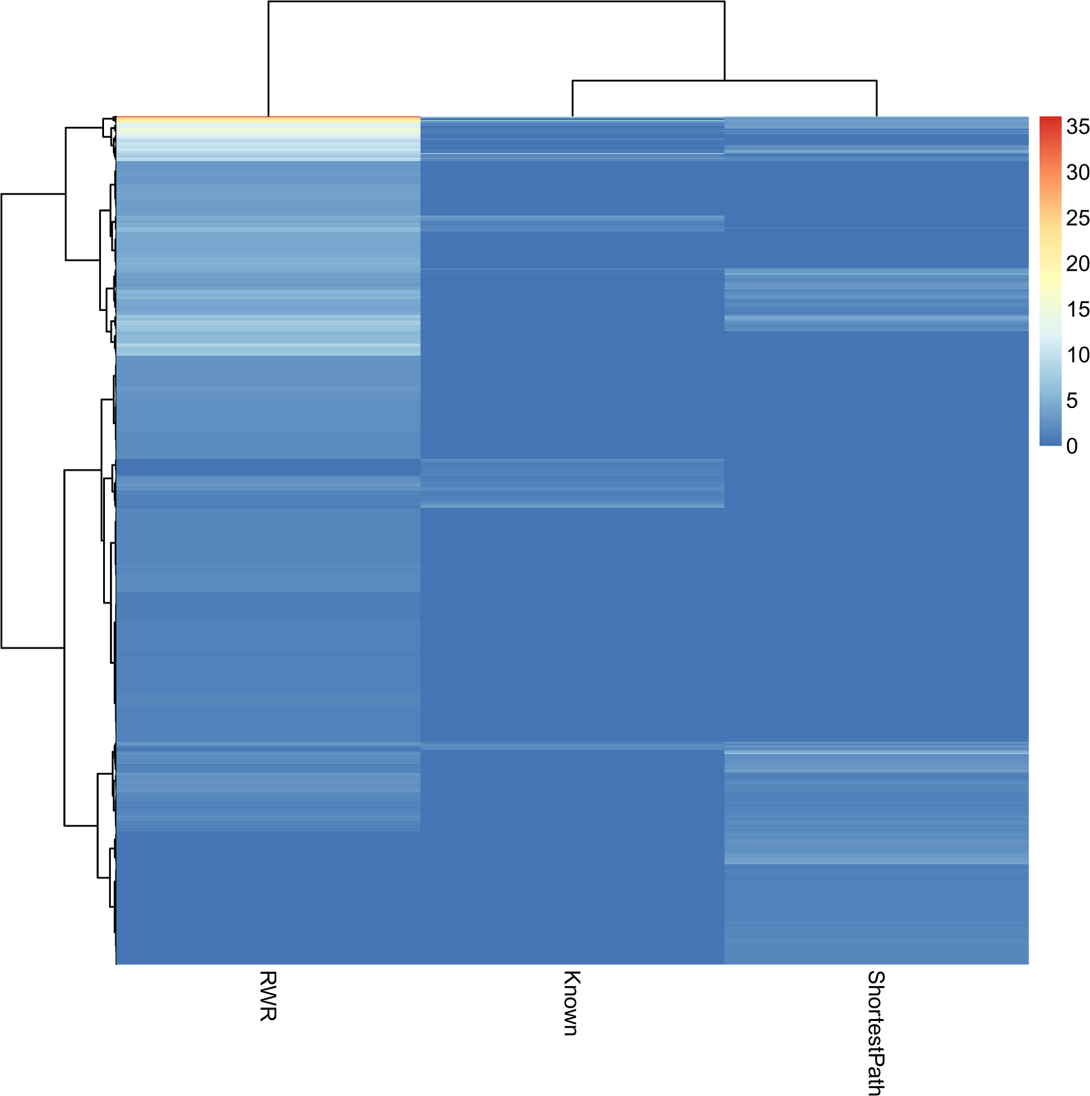
The heatmap of the functional profiles of three gene sets: known esophageal cancer related genes, shortest path predicted genes and RWR predicted genes. The color indicates the enrichment score, *i*.*e*.,-log10(enrichment p value). The shortest path predicted genes have more similar functions with the known esophageal cancer related genes as they are clustered together.

We did hierarchical clustering of these GO/KEGG terms/pathways and gene sets in **Fig 4**. It can be seen that the functional profile of shortest path predicted genes were more similar with known esophageal cancer related genes as the shortest path predicted genes and the known esophageal cancer related genes were clustered together.

Specifically, the top ten GO terms of known esophageal cancer related genes were enriched in the Wnt signaling pathway, cytoskeleton, cell response to steroid hormone stimulus, cell cycle regulation and cell death regulation. And the top ten GO terms of shortest path predicted genes were enriched in the Wnt signaling pathway, cytoskeleton and cellular response to stress, but the cell cycle and cell death regulation still have low p values. Meanwhile, the top ten GO terms of RWR predicted genes were enriched in the Wnt signaling pathway, regulation of cell death, regulation of cell proliferation, extracellular region part, epidermis development and ectoderm development. The esophagus develops from the human embryo which has three layers early in development. The endoderm forms the epithelial lining of the whole digestive tube except part of the mouth and pharynx and part of the rectum. During the second week, the embryo begins to surround and envelop portions of the embryonic yolk sac. Sections of the gut form organs of the gastrointestinal tract, including esophagus [112]. The eighth and ninth GO terms of the RWR predicted genes are epidermis development and ectoderm development, which is not reasonable.

Overall, the shortest path predicted genes were more likely to be novel esophageal cancer related genes based on their enriched functions.

## Conclusions

In this study, we generalized an existing computational method to identify new candidate genes and chemicals of esophageal cancer by constructing a hybrid network containing both proteins and chemicals. The results indicate that this method may help us identify novel genes and chemicals related to esophageal cancer. However, there are several limitations of this method. First, many other esophageal cancer factors, such as miRNAs and methylations were not considered in the analysis. If we can build a comprehensive integrative network which includes other factors, more novel genes and chemicals may be discovered. Secondly, we did not consider the molecular heterogeneity of esophageal cancer. If the subtype specific network, genes and chemicals are available, the model will be more precise. Third, with more computational power, the parameters of our method can be fine-tuned to achieve better performance.

## Supporting Information

S1 File 156 genes and 30 chemicals related to esophageal cancer (DOCX)

S1 Table 463 candidate genes and 90 candidate chemicals and their betweenness and permutation P-values (DOCX)

S2 Table 164 significant candidate genes and 24 significant candidate chemicals and their betweenness and permutation P-values (DOCX)

S3 Table The enrichments of 164 significant candidate genes on KEGG pathways (XLSX)

S4 Table The enrichments of 164 significant candidate genes on GO terms (XLSX)

S5 Table The enrichment score, *i*.*e*.,-log10(p value), of three gene sets: known esophageal cancer related genes, shortest path predicted genes and RWR predicted genes using DAVID (XLSX)
