# Supplementary material for: Identification of New Candidate Genes and Chemicals Related to Esophageal Cancer Using a Hybrid Interaction Network of Chemicals and Proteins
Source: PLoS One. 2015 Jun 9;10(6):e0129474. doi: 10.1371/journal.pone.0129474 (PMC4461353; doi:10.1371/journal.pone.0129474)
Supplement: S1 File — (DOCX) [file pone.0129474.s001.docx]

**S1 File.** 156 genes and 30 chemicals related to esophageal cancer

(I) 156 genes related to esophageal cancer

| APC | HTR | MET | PNN |
| --- | --- | --- | --- |
| TPR | WT1 | ADAMTS9 | ADAT2 |
| AGTPBP1 | AIFM2 | ALOX12 | ANAPC5 |
| ARL6IP6 | ARMC9 | ARMCX5 | ASCC1 |
| BRDT | C2orf40 | CAAP1 | CAPN13 |
| CASP10 | CD38 | CDH1 | CDKN1B |
| CDKN2A | CEACAM7 | CEP55 | CLCA2 |
| CLIC4 | COL19A1 | COL7A1 | CRIPAK |
| CRNN | CT45A5 | CTSB | CYP2C19 |
| 1-Dec | DERL1 | DKK4 | DLEC1 |
| DMTF1 | DPP4 | DSC3 | DSG1 |
| DSN1 | ECHDC3 | ENPP7 | EOGT |
| ERBB2 | ERO1L | ESYT2 | FANCD2 |
| FBXO4 | FGD5 | FGF8 | FHIT |
| FOLH1 | FZD1 | FZD10 | FZD2 |
| FZD4 | FZD5 | FZD7 | FZD9 |
| GALNT12 | GBP6 | GJB2 | GON4L |
| GRB7 | HEPHL1 | HJURP | HSPB1 |
| IL22RA1 | IRF1 | KLC4 | KLF6 |
| KRAS | KRT13 | KRT19 | KRT6A |
| KRT6B | KRT6C | KRT7 | KRT78 |
| LAMP3 | LATS2 | LCN2 | LITAF |
| LRRC16A | LRRC8E | LSP1 | LTA4H |
| LYPD3 | LZTFL1 | LZTS1 | MAGEA6 |
| MAGEA9 | MAPK6 | MMP10 | MMP13 |
| MSMB | MSR1 | MSRB3 | MUC4 |
| MUTYH | NFASC | NIPBL | NMES1 |
| ODC1 | P450 | PARN | PCNXL3 |
| PHF19 | PLS3 | PROM2 | PSCA |
| RAET1E | RHCG | RHOA | RNF6 |
| RTTN | SIRPB1 | SLC1A5 | SNX16 |
| SPINK7 | SRP72 | STARD3 | STK33 |
| SULF1 | SULF2 | TAP-1 | TCAIM |
| TFF1 | TGFBR2 | TP53 | TRAF4 |
| TRPV6 | TTBK1 | UIMC1 | USH2A |
| WWOX | XPNPEP3 | YAP1 | ZC3H14 |
| ZNF517 | ZNF766 | ANKRD19P | C11orf54 |
| KIAA2026 | KIDINS220 | SERPINB4 | SMARCAD1 |
| TBC1D22A | TMPRSS11A | TNFRSF10B | TNFRSF6B |

(II) 30 chemicals related to esophageal cancer

CID000000702 CID000002141 CID000002336 CID000002907

CID000003117 CID000003385 CID000003715 CID000005746

CID000005865 CID000005921 CID000005955 CID000005978

CID000006124 CID000007281 CID000007577 CID000008987

CID000010555 CID000013542 CID000023994 CID000031703

CID000041322 CID000041867 CID000060953 CID000072120

CID000088881 CID000148123 CID000445154 CID005280360

CID005351344 CID023690938
