# Supplementary material for: Identification of New Candidate Genes and Chemicals Related to Esophageal Cancer Using a Hybrid Interaction Network of Chemicals and Proteins
Source: PLoS One. 2015 Jun 9;10(6):e0129474. doi: 10.1371/journal.pone.0129474 (PMC4461353; doi:10.1371/journal.pone.0129474)
Supplement: S1 Table — (DOCX) [file pone.0129474.s002.docx]

**S1 Table.** 463 candidate genes and 90 candidate chemicals and their betweenness and permutation P-values

| **Protein or compound ID** | **Name** | **Betweenness** | **Permutation P-value** |
| --- | --- | --- | --- |
| ENSP00000364895 | ZBTB17 | 178 | <0.001 |
| ENSP00000284523 | WNT3A | 527 | <0.001 |
| ENSP00000166345 | TRIP13 | 178 | <0.001 |
| ENSP00000264731 | TP63 | 178 | <0.001 |
| ENSP00000343515 | TCEA2 | 178 | <0.001 |
| ENSP00000348128 | SVIL | 178 | <0.001 |
| ENSP00000216774 | SRP54 | 178 | <0.001 |
| ENSP00000230050 | RPS12 | 178 | <0.001 |
| ENSP00000361512 | PRPS1 | 178 | <0.001 |
| ENSP00000239940 | PFN2 | 178 | <0.001 |
| ENSP00000327801 | P4HB | 178 | <0.001 |
| ENSP00000338983 | MUC1 | 529 | <0.001 |
| ENSP00000313921 | MSRA | 178 | <0.001 |
| ENSP00000308208 | MMP14 | 178 | <0.001 |
| ENSP00000380504 | MEIOB | 178 | <0.001 |
| ENSP00000356070 | MAPKAPK2 | 354 | <0.001 |
| ENSP00000229794 | MAPK14 | 696 | <0.001 |
| ENSP00000261349 | LRP6 | 698 | <0.001 |
| ENSP00000338207 | LMO1 | 178 | <0.001 |
| ENSP00000381216 | KHSRP | 178 | <0.001 |
| ENSP00000349252 | ITGAL | 178 | <0.001 |
| ENSP00000262457 | INVS | 178 | <0.001 |
| ENSP00000329384 | IL22 | 178 | <0.001 |
| ENSP00000160262 | ICAM3 | 178 | <0.001 |
| ENSP00000380785 | HOOK2 | 1 | <0.001 |
| ENSP00000314080 | HIC1 | 178 | <0.001 |
| ENSP00000354900 | GJB1 | 178 | <0.001 |
| ENSP00000216336 | CTSG | 178 | <0.001 |
| ENSP00000344456 | CTNNB1 | 2294 | <0.001 |
| ENSP00000247306 | CTAG2 | 1 | <0.001 |
| ENSP00000353073 | CLN3 | 177 | <0.001 |
| ENSP00000316228 | CLEC4M | 178 | <0.001 |
| ENSP00000381522 | CHD9 | 178 | <0.001 |
| ENSP00000199764 | CEACAM6 | 178 | <0.001 |
| ENSP00000161559 | CEACAM1 | 178 | <0.001 |
| ENSP00000262320 | AXIN1 | 1063 | <0.001 |
| ENSP00000264110 | ATF2 | 428 | <0.001 |
| ENSP00000264448 | ALMS1 | 178 | <0.001 |
| ENSP00000359224 | ALG14 | 178 | <0.001 |
| ENSP00000361047 | ALG13 | 178 | <0.001 |
| ENSP00000391490 | AGR2 | 178 | <0.001 |
| ENSP00000361965 | ADA | 178 | <0.001 |
| ENSP00000341032 | WNT7B | 178 | 0.001 |
| ENSP00000296490 | WDR82 | 178 | 0.001 |
| ENSP00000315644 | TYMS | 527 | 0.001 |
| ENSP00000361311 | TMEM53 | 178 | 0.001 |
| ENSP00000354130 | SOX10 | 178 | 0.001 |
| ENSP00000262519 | SETD1A | 178 | 0.001 |
| ENSP00000296028 | PPBP | 3 | 0.001 |
| ENSP00000375921 | PAX3 | 178 | 0.001 |
| ENSP00000367462 | OLAH | 178 | 0.001 |
| ENSP00000278886 | NINL | 178 | 0.001 |
| ENSP00000311113 | JUP | 380 | 0.001 |
| ENSP00000342560 | HRH3 | 178 | 0.001 |
| ENSP00000356694 | FASLG | 178 | 0.001 |
| ENSP00000387006 | CWC22 | 178 | 0.001 |
| ENSP00000332018 | CTAG1A | 1 | 0.001 |
| ENSP00000264474 | CSTA | 178 | 0.001 |
| ENSP00000263168 | CAPZA1 | 178 | 0.001 |
| ENSP00000262584 | RPL8 | 178 | 0.002 |
| ENSP00000355652 | RHOU | 178 | 0.002 |
| ENSP00000327077 | PCM1 | 1 | 0.002 |
| ENSP00000262077 | NUP153 | 353 | 0.002 |
| ENSP00000239223 | DUSP1 | 100 | 0.002 |
| ENSP00000246891 | CSN1S1 | 178 | 0.002 |
| ENSP00000335325 | CCDC73 | 1 | 0.002 |
| ENSP00000242067 | BBS9 | 178 | 0.002 |
| ENSP00000278616 | ATM | 569 | 0.002 |
| ENSP00000368438 | PCNA | 1279 | 0.003 |
| ENSP00000345530 | NEDD4 | 178 | 0.003 |
| ENSP00000415183 | MUC2 | 178 | 0.003 |
| ENSP00000290200 | IL10RB | 178 | 0.003 |
| ENSP00000301838 | FADD | 332 | 0.003 |
| ENSP00000005340 | DVL2 | 351 | 0.003 |
| ENSP00000218388 | TIMP1 | 180 | 0.004 |
| ENSP00000240618 | KLRK1 | 178 | 0.004 |
| ENSP00000268057 | BBS4 | 1 | 0.004 |
| ENSP00000262238 | YY1 | 355 | 0.005 |
| ENSP00000245932 | VASP | 178 | 0.005 |
| ENSP00000263734 | EPAS1 | 17 | 0.005 |
| ENSP00000262768 | TIMP2 | 181 | 0.006 |
| ENSP00000260731 | KIF11 | 178 | 0.007 |
| ENSP00000227752 | IL10RA | 178 | 0.007 |
| ENSP00000412237 | IL10 | 178 | 0.007 |
| ENSP00000410294 | FGFR2 | 178 | 0.007 |
| ENSP00000353701 | DPP3 | 1 | 0.007 |
| ENSP00000242057 | AHR | 493 | 0.007 |
| ENSP00000265428 | WWP1 | 136 | 0.008 |
| ENSP00000369050 | CYP1A1 | 354 | 0.008 |
| ENSP00000351407 | ARNT | 485 | 0.008 |
| ENSP00000265441 | WNT2 | 177 | 0.009 |
| ENSP00000305459 | COG8 | 176 | 0.009 |
| ENSP00000371070 | ATP8A2 | 176 | 0.009 |
| ENSP00000265709 | ANK1 | 178 | 0.009 |
| ENSP00000358866 | FLNA | 178 | 0.01 |
| ENSP00000285018 | WNT7A | 1 | 0.011 |
| ENSP00000290158 | KPNB1 | 351 | 0.011 |
| ENSP00000335620 | GSTA1 | 242 | 0.011 |
| ENSP00000308021 | CEP290 | 178 | 0.011 |
| ENSP00000267082 | ITGB7 | 133 | 0.012 |
| ENSP00000241052 | CAT | 177 | 0.012 |
| ENSP00000337722 | ARL6 | 176 | 0.012 |
| ENSP00000332448 | ADAT3 | 2 | 0.012 |
| ENSP00000294339 | TAL1 | 178 | 0.014 |
| ENSP00000291700 | S100B | 178 | 0.014 |
| ENSP00000259808 | RIPK1 | 326 | 0.014 |
| ENSP00000297338 | RAD21 | 24 | 0.014 |
| ENSP00000364252 | PLA2G2A | 175 | 0.014 |
| ENSP00000257068 | MTNR1B | 6 | 0.014 |
| ENSP00000345344 | CTSL1 | 178 | 0.014 |
| ENSP00000270349 | SLC6A3 | 178 | 0.015 |
| ENSP00000219070 | MMP2 | 228 | 0.015 |
| ENSP00000304592 | FASN | 178 | 0.015 |
| ENSP00000228837 | FGF6 | 178 | 0.016 |
| ENSP00000336868 | CENPA | 178 | 0.017 |
| ENSP00000265517 | MTTP | 5 | 0.018 |
| ENSP00000359211 | DPYD | 177 | 0.018 |
| ENSP00000262965 | TCF3 | 178 | 0.019 |
| ENSP00000276297 | DLC1 | 175 | 0.019 |
| ENSP00000361405 | MMP9 | 186 | 0.02 |
| ENSP00000264634 | WNT5A | 1 | 0.021 |
| ENSP00000408526 | IMPA1 | 39 | 0.021 |
| ENSP00000292853 | FBXO27 | 174 | 0.021 |
| ENSP00000323967 | SMARCE1 | 177 | 0.022 |
| ENSP00000359074 | L1CAM | 178 | 0.022 |
| ENSP00000382133 | DNA2 | 177 | 0.023 |
| ENSP00000371634 | IGF2BP2 | 171 | 0.024 |
| ENSP00000255030 | CRP | 177 | 0.025 |
| ENSP00000312455 | CFLAR | 84 | 0.025 |
| ENSP00000296674 | RPS23 | 77 | 0.027 |
| ENSP00000302021 | MUC7 | 1 | 0.028 |
| ENSP00000211122 | GSTA3 | 1 | 0.028 |
| ENSP00000351997 | MAP2K6 | 141 | 0.029 |
| ENSP00000233146 | MSH2 | 178 | 0.03 |
| ENSP00000314004 | ANAPC2 | 178 | 0.03 |
| ENSP00000361433 | EXOSC2 | 3 | 0.031 |
| ENSP00000262367 | CREBBP | 744 | 0.031 |
| ENSP00000303315 | JUNB | 5 | 0.032 |
| ENSP00000252945 | CYP2E1 | 179 | 0.032 |
| ENSP00000354612 | PTGS1 | 182 | 0.033 |
| ENSP00000376776 | DBH | 173 | 0.033 |
| ENSP00000360025 | GADD45A | 102 | 0.034 |
| ENSP00000245157 | BBS2 | 177 | 0.034 |
| ENSP00000317955 | EEA1 | 3 | 0.035 |
| ENSP00000351486 | NTRK1 | 302 | 0.036 |
| ENSP00000217244 | CSNK2A1 | 178 | 0.036 |
| ENSP00000360266 | JUN | 901 | 0.037 |
| ENSP00000311469 | GSTM1 | 2 | 0.039 |
| ENSP00000346839 | FN1 | 581 | 0.039 |
| ENSP00000379339 | RPS29 | 101 | 0.04 |
| ENSP00000278568 | PAK1 | 182 | 0.04 |
| ENSP00000377865 | RPL23 | 1 | 0.041 |
| ENSP00000305480 | FEN1 | 177 | 0.041 |
| ENSP00000355536 | MTR | 308 | 0.042 |
| ENSP00000258654 | COG3 | 176 | 0.043 |
| ENSP00000359300 | CETN2 | 178 | 0.043 |
| ENSP00000358105 | APH1A | 13 | 0.043 |
| ENSP00000300289 | PDIA3 | 140 | 0.044 |
| ENSP00000382342 | ABCC1 | 169 | 0.044 |
| ENSP00000252029 | TYMP | 6 | 0.045 |
| ENSP00000359939 | EXOSC1 | 1 | 0.045 |
| ENSP00000010338 | TRAF3IP3 | 175 | 0.047 |
| ENSP00000293379 | ITGA5 | 369 | 0.048 |
| ENSP00000364094 | ITGB1 | 418 | 0.049 |
| ENSP00000302955 | RRM2 | 27 | 0.05 |
| ENSP00000351777 | VCP | 178 | 0.051 |
| ENSP00000340937 | COL17A1 | 95 | 0.051 |
| ENSP00000288986 | NCK1 | 134 | 0.052 |
| ENSP00000338297 | IGF2 | 173 | 0.052 |
| ENSP00000354586 | GLI2 | 177 | 0.052 |
| ENSP00000358335 | MAP3K7 | 141 | 0.053 |
| ENSP00000344352 | ATF3 | 178 | 0.054 |
| ENSP00000355629 | COG2 | 176 | 0.056 |
| ENSP00000262629 | TYROBP | 354 | 0.058 |
| ENSP00000367316 | ITGA8 | 146 | 0.059 |
| ENSP00000401303 | SHC1 | 286 | 0.06 |
| ENSP00000411532 | TOP2A | 176 | 0.061 |
| ENSP00000269349 | EIF4A3 | 178 | 0.061 |
| ENSP00000300161 | YWHAB | 153 | 0.062 |
| ENSP00000256996 | DDB2 | 9 | 0.062 |
| ENSP00000346389 | MEF2A | 2 | 0.063 |
| ENSP00000381331 | HDAC2 | 223 | 0.064 |
| ENSP00000360683 | PTPN1 | 189 | 0.065 |
| ENSP00000353483 | MAPK8 | 257 | 0.065 |
| ENSP00000358727 | GSTO1 | 1 | 0.065 |
| ENSP00000285021 | XPC | 178 | 0.067 |
| ENSP00000200181 | ITGB4 | 95 | 0.068 |
| ENSP00000380227 | ITGA4 | 147 | 0.068 |
| ENSP00000359998 | GSTA4 | 2 | 0.068 |
| ENSP00000256442 | CCNB1 | 1059 | 0.068 |
| ENSP00000172229 | NGFR | 178 | 0.069 |
| ENSP00000311032 | CASP3 | 295 | 0.069 |
| ENSP00000302530 | BUB1 | 404 | 0.07 |
| ENSP00000256474 | VHL | 559 | 0.072 |
| ENSP00000369871 | HAUS6 | 177 | 0.074 |
| ENSP00000332643 | NDN | 18 | 0.077 |
| ENSP00000315859 | RNPS1 | 178 | 0.081 |
| ENSP00000296585 | ITGA2 | 198 | 0.083 |
| ENSP00000245960 | CDC25B | 47 | 0.084 |
| ENSP00000226218 | SEBOX | 8 | 0.085 |
| ENSP00000419692 | RXRA | 182 | 0.085 |
| ENSP00000362361 | CDK9 | 178 | 0.085 |
| ENSP00000352798 | COL18A1 | 81 | 0.086 |
| ENSP00000340820 | MAPT | 178 | 0.092 |
| ENSP00000256857 | GRP | 176 | 0.093 |
| ENSP00000386896 | ITGA6 | 95 | 0.098 |
| ENSP00000398698 | TNF | 177 | 0.099 |
| ENSP00000380280 | FGFR1 | 178 | 0.099 |
| ENSP00000364898 | SYK | 353 | 0.102 |
| ENSP00000316460 | FYB | 22 | 0.102 |
| ENSP00000269141 | CDH2 | 539 | 0.103 |
| ENSP00000247461 | CANX | 140 | 0.103 |
| ENSP00000231487 | SKP1 | 266 | 0.104 |
| ENSP00000297268 | COL1A2 | 1 | 0.105 |
| ENSP00000364133 | TGFBR1 | 330 | 0.106 |
| ENSP00000257181 | PRPF38A | 83 | 0.106 |
| ENSP00000338018 | HIF1A | 915 | 0.106 |
| ENSP00000363512 | ALOX5 | 6 | 0.107 |
| ENSP00000303242 | ITGB2 | 177 | 0.11 |
| ENSP00000251968 | TSG101 | 178 | 0.111 |
| ENSP00000400591 | SNRPE | 18 | 0.111 |
| ENSP00000261707 | SLC6A4 | 17 | 0.111 |
| ENSP00000264515 | RBBP5 | 5 | 0.111 |
| ENSP00000351273 | CASP8 | 115 | 0.112 |
| ENSP00000261597 | NDC80 | 177 | 0.114 |
| ENSP00000046794 | LCP2 | 65 | 0.114 |
| ENSP00000396127 | RAN | 351 | 0.117 |
| ENSP00000264832 | ICAM1 | 1 | 0.117 |
| ENSP00000358918 | SUFU | 1 | 0.118 |
| ENSP00000268035 | IGF1R | 350 | 0.118 |
| ENSP00000261900 | CCNT1 | 1 | 0.118 |
| ENSP00000262158 | SMAD7 | 220 | 0.119 |
| ENSP00000348577 | RANGAP1 | 345 | 0.122 |
| ENSP00000260363 | KIF23 | 4 | 0.122 |
| ENSP00000260682 | CYP2C9 | 10 | 0.124 |
| ENSP00000343204 | JAK1 | 178 | 0.132 |
| ENSP00000227758 | BIRC2 | 141 | 0.134 |
| ENSP00000225964 | COL1A1 | 177 | 0.136 |
| ENSP00000354859 | DRD2 | 50 | 0.137 |
| ENSP00000273047 | RAB5A | 3 | 0.138 |
| ENSP00000250003 | MYOD1 | 178 | 0.138 |
| ENSP00000351446 | WDR5 | 173 | 0.14 |
| ENSP00000338345 | SNCA | 139 | 0.141 |
| ENSP00000358301 | ADRB1 | 2 | 0.142 |
| ENSP00000379110 | CXCL1 | 1 | 0.144 |
| ENSP00000363092 | PRKG1 | 3 | 0.145 |
| ENSP00000265724 | ABCB1 | 16 | 0.154 |
| ENSP00000371973 | SAP18 | 1 | 0.155 |
| ENSP00000321656 | CDC25C | 6 | 0.159 |
| ENSP00000009589 | RPS20 | 1 | 0.16 |
| ENSP00000297261 | SHH | 176 | 0.162 |
| ENSP00000326804 | CUL1 | 174 | 0.163 |
| ENSP00000260970 | PPIG | 83 | 0.166 |
| ENSP00000324648 | CYP2B6 | 13 | 0.167 |
| ENSP00000316244 | HTR1A | 127 | 0.17 |
| ENSP00000233242 | APOB | 5 | 0.171 |
| ENSP00000211998 | VCL | 7 | 0.172 |
| ENSP00000320866 | CALR | 171 | 0.172 |
| ENSP00000162749 | TNFRSF1A | 177 | 0.173 |
| ENSP00000229022 | VDR | 215 | 0.175 |
| ENSP00000356087 | IKBKE | 11 | 0.175 |
| ENSP00000344818 | UBC | 6250 | 0.177 |
| ENSP00000319635 | CXCR2 | 148 | 0.18 |
| ENSP00000324806 | GSK3B | 195 | 0.184 |
| ENSP00000287820 | PPARG | 274 | 0.187 |
| ENSP00000227507 | CCND1 | 546 | 0.188 |
| ENSP00000251849 | RAF1 | 844 | 0.192 |
| ENSP00000353059 | APAF1 | 109 | 0.192 |
| ENSP00000370473 | IGFBP3 | 207 | 0.194 |
| ENSP00000262435 | SMURF2 | 1 | 0.197 |
| ENSP00000250495 | NEDD8 | 39 | 0.202 |
| ENSP00000291552 | U2AF1 | 81 | 0.204 |
| ENSP00000362900 | SRSF4 | 2 | 0.204 |
| ENSP00000358541 | SIKE1 | 11 | 0.204 |
| ENSP00000332353 | PTCH1 | 176 | 0.205 |
| ENSP00000354720 | SMC3 | 154 | 0.207 |
| ENSP00000302961 | HSPA4 | 8 | 0.208 |
| ENSP00000275493 | EGFR | 386 | 0.213 |
| ENSP00000244007 | PLCG1 | 180 | 0.217 |
| ENSP00000350720 | SMARCA4 | 177 | 0.22 |
| ENSP00000297151 | SLU7 | 83 | 0.221 |
| ENSP00000351894 | NCOA6 | 5 | 0.226 |
| ENSP00000359345 | RPL5 | 277 | 0.227 |
| ENSP00000365775 | MTHFR | 1 | 0.227 |
| ENSP00000302486 | MAP2K1 | 143 | 0.228 |
| ENSP00000237612 | ABCG2 | 3 | 0.229 |
| ENSP00000324804 | PPP2R1A | 24 | 0.24 |
| ENSP00000396308 | DHFR | 3 | 0.243 |
| ENSP00000261023 | ITGAV | 8 | 0.244 |
| ENSP00000316879 | EIF4G1 | 163 | 0.244 |
| ENSP00000339151 | IKBKB | 2 | 0.245 |
| ENSP00000320940 | NCOA1 | 515 | 0.248 |
| ENSP00000338934 | EZR | 178 | 0.25 |
| ENSP00000359531 | GTF2B | 25 | 0.256 |
| ENSP00000359206 | BTRC | 2 | 0.258 |
| ENSP00000306512 | IL8 | 146 | 0.259 |
| ENSP00000260433 | CYP19A1 | 80 | 0.263 |
| ENSP00000278916 | CHEK1 | 10 | 0.264 |
| ENSP00000354394 | STAT1 | 177 | 0.265 |
| ENSP00000305372 | ADRB2 | 251 | 0.266 |
| ENSP00000337088 | MEN1 | 157 | 0.268 |
| ENSP00000361066 | NCOA3 | 12 | 0.272 |
| ENSP00000302665 | IGF1 | 197 | 0.278 |
| ENSP00000359727 | BAG2 | 83 | 0.281 |
| ENSP00000252818 | JUND | 157 | 0.282 |
| ENSP00000337915 | CYP3A4 | 354 | 0.286 |
| ENSP00000221930 | TGFB1 | 171 | 0.291 |
| ENSP00000356438 | PTGS2 | 159 | 0.293 |
| ENSP00000341344 | GGA1 | 3 | 0.295 |
| ENSP00000011653 | CD4 | 177 | 0.296 |
| ENSP00000324897 | UBE2I | 328 | 0.298 |
| ENSP00000382004 | CTNND1 | 30 | 0.302 |
| ENSP00000350708 | RAD23B | 169 | 0.304 |
| ENSP00000312995 | CLSPN | 4 | 0.307 |
| ENSP00000264033 | CBL | 1182 | 0.307 |
| ENSP00000378529 | FZR1 | 7 | 0.308 |
| ENSP00000313950 | AURKB | 178 | 0.309 |
| ENSP00000262613 | SLC9A3R1 | 177 | 0.31 |
| ENSP00000216225 | RBX1 | 103 | 0.312 |
| ENSP00000295897 | ALB | 140 | 0.312 |
| ENSP00000269321 | ARHGDIA | 55 | 0.315 |
| ENSP00000301764 | DDB1 | 9 | 0.321 |
| ENSP00000354522 | TOP1 | 3 | 0.324 |
| ENSP00000310127 | IRF3 | 11 | 0.324 |
| ENSP00000349437 | IGF2R | 3 | 0.326 |
| ENSP00000280892 | EIF4E | 163 | 0.327 |
| ENSP00000223095 | SERPINE1 | 8 | 0.33 |
| ENSP00000350275 | HIST1H3A | 16 | 0.333 |
| ENSP00000257904 | CDK4 | 32 | 0.337 |
| ENSP00000216797 | NFKBIA | 2 | 0.355 |
| ENSP00000274255 | SKP2 | 123 | 0.357 |
| ENSP00000263253 | EP300 | 447 | 0.357 |
| ENSP00000300134 | STAT6 | 4 | 0.358 |
| ENSP00000345571 | E2F1 | 18 | 0.359 |
| ENSP00000216605 | MTHFD1 | 1 | 0.365 |
| ENSP00000281708 | FBXW7 | 11 | 0.366 |
| ENSP00000342007 | CYP1A2 | 2 | 0.368 |
| ENSP00000231509 | NR3C1 | 185 | 0.37 |
| ENSP00000261461 | PPP2R5A | 24 | 0.372 |
| ENSP00000367207 | MYC | 179 | 0.374 |
| ENSP00000219548 | STUB1 | 148 | 0.377 |
| ENSP00000357879 | PSMD4 | 169 | 0.379 |
| ENSP00000417281 | MDM2 | 538 | 0.382 |
| ENSP00000215587 | POLR2E | 2 | 0.386 |
| ENSP00000244741 | CDKN1A | 27 | 0.392 |
| ENSP00000258962 | SRSF1 | 9 | 0.393 |
| ENSP00000228307 | PXN | 46 | 0.4 |
| ENSP00000314949 | POLR2A | 177 | 0.401 |
| ENSP00000215832 | MAPK1 | 163 | 0.401 |
| ENSP00000262477 | RABEP1 | 3 | 0.404 |
| ENSP00000242577 | DYNLL1 | 55 | 0.406 |
| ENSP00000363676 | RPL11 | 252 | 0.412 |
| ENSP00000354558 | MTOR | 161 | 0.415 |
| ENSP00000384273 | RELA | 157 | 0.417 |
| ENSP00000352516 | DNMT1 | 291 | 0.428 |
| ENSP00000236850 | APOA1 | 8 | 0.433 |
| ENSP00000361423 | ABL1 | 47 | 0.441 |
| ENSP00000209728 | CDC6 | 43 | 0.443 |
| ENSP00000304895 | IRS1 | 154 | 0.447 |
| ENSP00000249299 | NAA38 | 3 | 0.451 |
| ENSP00000362820 | SRSF3 | 2 | 0.459 |
| ENSP00000249071 | RAC2 | 41 | 0.474 |
| ENSP00000262643 | CCNE1 | 45 | 0.474 |
| ENSP00000308450 | CDC20 | 109 | 0.48 |
| ENSP00000310596 | LSM1 | 6 | 0.481 |
| ENSP00000332973 | SMAD3 | 1 | 0.488 |
| ENSP00000342952 | ADCY2 | 39 | 0.491 |
| ENSP00000267163 | RB1 | 288 | 0.497 |
| ENSP00000264381 | BCHE | 178 | 0.497 |
| ENSP00000337825 | LCK | 181 | 0.503 |
| ENSP00000299421 | ILK | 1 | 0.507 |
| ENSP00000230354 | TBP | 54 | 0.511 |
| ENSP00000350877 | SRSF2 | 2 | 0.513 |
| ENSP00000376076 | SUMO1 | 17 | 0.514 |
| ENSP00000358525 | NGF | 58 | 0.522 |
| ENSP00000247668 | TRAF2 | 166 | 0.529 |
| ENSP00000302564 | BCL2L1 | 2 | 0.529 |
| ENSP00000302967 | HDAC3 | 2 | 0.531 |
| ENSP00000300574 | CRK | 19 | 0.544 |
| ENSP00000268712 | NCOR1 | 14 | 0.554 |
| ENSP00000362649 | HDAC1 | 308 | 0.554 |
| ENSP00000280097 | HNMT | 5 | 0.558 |
| ENSP00000369757 | RPS6 | 1 | 0.564 |
| ENSP00000261799 | PDGFRB | 29 | 0.564 |
| ENSP00000374354 | EXOSC8 | 2 | 0.567 |
| ENSP00000308938 | PLG | 39 | 0.57 |
| ENSP00000329623 | BCL2 | 31 | 0.577 |
| ENSP00000266970 | CDK2 | 230 | 0.582 |
| ENSP00000299543 | CTDP1 | 6 | 0.589 |
| ENSP00000300093 | PLK1 | 225 | 0.591 |
| ENSP00000219476 | TSC2 | 9 | 0.601 |
| ENSP00000274376 | RASA1 | 28 | 0.611 |
| ENSP00000358022 | MCL1 | 22 | 0.612 |
| ENSP00000329357 | SP1 | 21 | 0.621 |
| ENSP00000340944 | PTPN11 | 7 | 0.623 |
| ENSP00000350283 | BRCA1 | 197 | 0.625 |
| ENSP00000268058 | PML | 17 | 0.631 |
| ENSP00000254066 | RARA | 17 | 0.635 |
| ENSP00000262160 | SMAD2 | 12 | 0.639 |
| ENSP00000312735 | POLR2B | 74 | 0.641 |
| ENSP00000227378 | HSPA8 | 83 | 0.643 |
| ENSP00000294172 | NXF1 | 2 | 0.644 |
| ENSP00000276201 | UPF3B | 9 | 0.655 |
| ENSP00000384675 | SOS1 | 157 | 0.656 |
| ENSP00000264708 | POMC | 2 | 0.66 |
| ENSP00000264657 | STAT3 | 154 | 0.666 |
| ENSP00000418447 | PPP2CA | 85 | 0.669 |
| ENSP00000263753 | SGOL1 | 77 | 0.674 |
| ENSP00000340691 | EIF4EBP1 | 1 | 0.681 |
| ENSP00000252622 | LSM7 | 26 | 0.684 |
| ENSP00000300651 | MED1 | 10 | 0.685 |
| ENSP00000303830 | INSR | 77 | 0.687 |
| ENSP00000326366 | PSEN1 | 13 | 0.692 |
| ENSP00000302269 | VAV1 | 2 | 0.711 |
| ENSP00000348986 | INS-IGF2 | 77 | 0.712 |
| ENSP00000306245 | FOS | 128 | 0.725 |
| ENSP00000350941 | SRC | 332 | 0.729 |
| ENSP00000340330 | KAT5 | 3 | 0.739 |
| ENSP00000262803 | UPF1 | 2 | 0.749 |
| ENSP00000287936 | HMGCR | 172 | 0.757 |
| ENSP00000264951 | XRN1 | 1 | 0.758 |
| ENSP00000363822 | AR | 92 | 0.771 |
| ENSP00000348708 | UPF2 | 8 | 0.777 |
| ENSP00000414634 | LSM2 | 7 | 0.781 |
| ENSP00000309103 | BAD | 2 | 0.788 |
| ENSP00000340858 | B2M | 28 | 0.792 |
| ENSP00000417404 | HFE | 16 | 0.794 |
| ENSP00000353224 | TFRC | 16 | 0.797 |
| ENSP00000348461 | RAC1 | 11 | 0.803 |
| ENSP00000309845 | HRAS | 862 | 0.806 |
| ENSP00000287598 | BUB1B | 80 | 0.815 |
| ENSP00000215829 | SNRPD3 | 31 | 0.82 |
| ENSP00000309503 | YWHAZ | 2 | 0.829 |
| ENSP00000366135 | EXOSC10 | 126 | 0.829 |
| ENSP00000371067 | JAK2 | 5 | 0.834 |
| ENSP00000162330 | BCAR1 | 22 | 0.836 |
| ENSP00000339109 | ANAPC1 | 1 | 0.849 |
| ENSP00000240185 | TARDBP | 126 | 0.857 |
| ENSP00000248566 | SHFM1 | 75 | 0.861 |
| ENSP00000263967 | PIK3CA | 141 | 0.862 |
| ENSP00000307863 | U2AF2 | 112 | 0.863 |
| ENSP00000369497 | BRCA2 | 1 | 0.872 |
| ENSP00000263309 | CLNS1A | 23 | 0.875 |
| ENSP00000357656 | FYN | 6 | 0.877 |
| ENSP00000319169 | PRMT5 | 23 | 0.878 |
| ENSP00000365891 | WAS | 4 | 0.88 |
| ENSP00000274026 | CCNA2 | 181 | 0.882 |
| ENSP00000206249 | ESR1 | 1288 | 0.911 |
| ENSP00000235090 | WDR77 | 23 | 0.912 |
| ENSP00000378165 | ZNF207 | 23 | 0.913 |
| ENSP00000221494 | SF3A2 | 38 | 0.916 |
| ENSP00000357858 | BUB3 | 23 | 0.917 |
| ENSP00000365439 | HNRNPK | 112 | 0.924 |
| ENSP00000341189 | PTK2 | 65 | 0.925 |
| ENSP00000335153 | HSP90AA1 | 1790 | 0.93 |
| ENSP00000270202 | AKT1 | 273 | 0.934 |
| ENSP00000274335 | PIK3R1 | 55 | 0.937 |
| ENSP00000313829 | KHDRBS1 | 112 | 0.943 |
| ENSP00000223023 | WASL | 14 | 0.948 |
| ENSP00000339007 | GRB2 | 213 | 0.952 |
| ENSP00000292644 | PSMC2 | 72 | 0.959 |
| ENSP00000314458 | CDC42 | 64 | 0.968 |
| ENSP00000003084 | CFTR | 1344 | 0.988 |
| CID000157350 | hydroxyl radical | 178 | <0.001 |
| CID000010461 | sulfur mustard | 354 | <0.001 |
| CID000031356 | Tris-BP | 177 | 0.001 |
| CID000004908 | primaquine | 177 | 0.001 |
| CID005324345 | fluvoxamine | 2 | 0.002 |
| CID000440399 | toluene cis-dihydrodiol | 177 | 0.002 |
| CID000082313 | alpha-GlcNAc | 177 | 0.002 |
| CID000002771 | citalopram | 16 | 0.002 |
| CID000002733 | chlorzoxazone | 4 | 0.006 |
| CID000001014 | phosphorylcholine | 178 | 0.006 |
| CID000439655 | d-tartrate | 44 | 0.007 |
| CID000001117 | sulfate | 178 | 0.012 |
| CID000520535 | superoxide | 178 | 0.017 |
| CID000004174 | metyrapone | 7 | 0.017 |
| CID000072886 | L-cysteate | 178 | 0.022 |
| CID000001140 | toluene | 177 | 0.022 |
| CID000119400 | tartrate | 44 | 0.028 |
| CID000000019 | 2,3-dihydroxybenzoic a | 2 | 0.032 |
| CID011020241 | ascorbate | 1 | 0.038 |
| CID000105024 | L-selenomethionine | 311 | 0.039 |
| CID000001775 | phenytoin | 10 | 0.04 |
| CID000003016 | diazepam | 112 | 0.044 |
| CID000030323 | anthracycline | 2 | 0.046 |
| CID000000784 | H2O2 | 353 | 0.046 |
| CID000005472 | ticlopidine | 16 | 0.05 |
| CID000001983 | acetaminophen | 2 | 0.052 |
| CID000006804 | 5'-GMP | 178 | 0.054 |
| CID000005789 | thymidine | 5 | 0.064 |
| CID000006021 | inosine | 2 | 0.069 |
| CID000006083 | adenosine monophosphate | 358 | 0.071 |
| CID000000222 | ammonia | 223 | 0.071 |
| CID000000177 | acetaldehyde | 189 | 0.078 |
| CID000000936 | vitamin B | 1 | 0.086 |
| CID000010238 | adenosine 5'-phosphosulfate | 3 | 0.095 |
| CID000445675 | UDP-GlcNAc | 178 | 0.102 |
| CID000005881 | DHEA | 2 | 0.11 |
| CID000000896 | melatonin | 6 | 0.112 |
| CID000001102 | spermidine | 174 | 0.113 |
| CID000033032 | glutamic a | 351 | 0.12 |
| CID005280453 | 1,25(OH)2D3 | 177 | 0.122 |
| CID000003032 | diclofenac | 27 | 0.13 |
| CID000446220 | cocaine | 10 | 0.138 |
| CID000003779 | isoproterenol | 4 | 0.145 |
| CID000002520 | verapamil | 11 | 0.152 |
| CID000004828 | pindolol | 125 | 0.155 |
| CID000006076 | cyclic AMP | 178 | 0.157 |
| CID000002244 | aspirin | 4 | 0.171 |
| CID000000402 | sulfur | 66 | 0.178 |
| CID000002818 | clozapine | 22 | 0.223 |
| CID000060699 | topotecan | 3 | 0.231 |
| CID000004763 | phenobarbital | 9 | 0.231 |
| CID000124886 | Glutathione | 106 | 0.232 |
| CID000024139 | chitin | 179 | 0.235 |
| CID000439260 | norepinephrine | 176 | 0.247 |
| CID000000977 | oxygen | 892 | 0.249 |
| CID000024316 | cyclic GMP | 3 | 0.256 |
| CID000060961 | adenosine | 177 | 0.26 |
| CID000000774 | histamine | 201 | 0.311 |
| CID000066370 | alpha-D-glucose | 2 | 0.33 |
| CID000000991 | parathion | 178 | 0.34 |
| CID000439155 | AdoHcy | 307 | 0.358 |
| CID002733525 | tamoxifen | 330 | 0.361 |
| CID000005892 | beta-NAD | 360 | 0.408 |
| CID000000681 | dopamine | 83 | 0.419 |
| CID000004585 | olanzapine | 4 | 0.467 |
| CID000444899 | arachidonic a | 61 | 0.485 |
| CID000001935 | tacrine | 7 | 0.491 |
| CID000000190 | adenine | 5 | 0.539 |
| CID000006022 | adenosine diphosphate | 198 | 0.541 |
| CID000006013 | testosterone | 80 | 0.544 |
| CID000005202 | serotonin | 6 | 0.568 |
| CID000005997 | cholesterol | 176 | 0.573 |
| CID000064689 | D-glucose | 2 | 0.618 |
| CID000000312 | chloride | 297 | 0.627 |
| CID000444493 | acetyl-CoA | 5 | 0.638 |
| CID000000679 | DMSO | 6 | 0.657 |
| CID000006031 | uridine diphosphate | 7 | 0.692 |
| CID000000305 | choline | 171 | 0.75 |
| CID000005885 | NADP | 146 | 0.799 |
| CID000000271 | calcium | 2098 | 0.843 |
| CID000008977 | guanosine diphosphate | 182 | 0.885 |
| CID000023925 | Fe(III | 88 | 0.892 |
| CID000006830 | guanosine triphosphate | 624 | 0.905 |
| CID000087642 | coenzyme A | 4 | 0.921 |
| CID000000753 | glycerol | 275 | 0.943 |
| CID000000888 | magnesium | 140 | 0.98 |
| CID000000051 | alpha-ketoglutarate | 1 | 0.981 |
| CID000000923 | sodium | 48 | 0.987 |
| CID000000961 | hydroxyl radicals | 8 | 0.989 |
| CID000005957 | adenosine triphosphate | 2900 | 1 |
