# Supplementary material for: Identification of New Candidate Genes and Chemicals Related to Esophageal Cancer Using a Hybrid Interaction Network of Chemicals and Proteins
Source: PLoS One. 2015 Jun 9;10(6):e0129474. doi: 10.1371/journal.pone.0129474 (PMC4461353; doi:10.1371/journal.pone.0129474)
Supplement: S2 Table — (DOCX) [file pone.0129474.s003.docx]

**S2 Table.** 164 significant candidate genes and 24 significant candidate chemicals and their betweenness and permutation P-values

| **Protein or compound ID** | **Name** | **Betweenness** | **Permutation P-value** |
| --- | --- | --- | --- |
| ENSP00000364895 | ZBTB17 | 178 | <0.001 |
| ENSP00000284523 | WNT3A | 527 | <0.001 |
| ENSP00000166345 | TRIP13 | 178 | <0.001 |
| ENSP00000264731 | TP63 | 178 | <0.001 |
| ENSP00000343515 | TCEA2 | 178 | <0.001 |
| ENSP00000348128 | SVIL | 178 | <0.001 |
| ENSP00000216774 | SRP54 | 178 | <0.001 |
| ENSP00000230050 | RPS12 | 178 | <0.001 |
| ENSP00000361512 | PRPS1 | 178 | <0.001 |
| ENSP00000239940 | PFN2 | 178 | <0.001 |
| ENSP00000327801 | P4HB | 178 | <0.001 |
| ENSP00000338983 | MUC1 | 529 | <0.001 |
| ENSP00000313921 | MSRA | 178 | <0.001 |
| ENSP00000308208 | MMP14 | 178 | <0.001 |
| ENSP00000380504 | MEIOB | 178 | <0.001 |
| ENSP00000356070 | MAPKAPK2 | 354 | <0.001 |
| ENSP00000229794 | MAPK14 | 696 | <0.001 |
| ENSP00000261349 | LRP6 | 698 | <0.001 |
| ENSP00000338207 | LMO1 | 178 | <0.001 |
| ENSP00000381216 | KHSRP | 178 | <0.001 |
| ENSP00000349252 | ITGAL | 178 | <0.001 |
| ENSP00000262457 | INVS | 178 | <0.001 |
| ENSP00000329384 | IL22 | 178 | <0.001 |
| ENSP00000160262 | ICAM3 | 178 | <0.001 |
| ENSP00000380785 | HOOK2 | 1 | <0.001 |
| ENSP00000314080 | HIC1 | 178 | <0.001 |
| ENSP00000354900 | GJB1 | 178 | <0.001 |
| ENSP00000216336 | CTSG | 178 | <0.001 |
| ENSP00000344456 | CTNNB1 | 2294 | <0.001 |
| ENSP00000247306 | CTAG2 | 1 | <0.001 |
| ENSP00000353073 | CLN3 | 177 | <0.001 |
| ENSP00000316228 | CLEC4M | 178 | <0.001 |
| ENSP00000381522 | CHD9 | 178 | <0.001 |
| ENSP00000199764 | CEACAM6 | 178 | <0.001 |
| ENSP00000161559 | CEACAM1 | 178 | <0.001 |
| ENSP00000262320 | AXIN1 | 1063 | <0.001 |
| ENSP00000264110 | ATF2 | 428 | <0.001 |
| ENSP00000264448 | ALMS1 | 178 | <0.001 |
| ENSP00000359224 | ALG14 | 178 | <0.001 |
| ENSP00000361047 | ALG13 | 178 | <0.001 |
| ENSP00000391490 | AGR2 | 178 | <0.001 |
| ENSP00000361965 | ADA | 178 | <0.001 |
| ENSP00000341032 | WNT7B | 178 | 0.001 |
| ENSP00000296490 | WDR82 | 178 | 0.001 |
| ENSP00000315644 | TYMS | 527 | 0.001 |
| ENSP00000361311 | TMEM53 | 178 | 0.001 |
| ENSP00000354130 | SOX10 | 178 | 0.001 |
| ENSP00000262519 | SETD1A | 178 | 0.001 |
| ENSP00000296028 | PPBP | 3 | 0.001 |
| ENSP00000375921 | PAX3 | 178 | 0.001 |
| ENSP00000367462 | OLAH | 178 | 0.001 |
| ENSP00000278886 | NINL | 178 | 0.001 |
| ENSP00000311113 | JUP | 380 | 0.001 |
| ENSP00000342560 | HRH3 | 178 | 0.001 |
| ENSP00000356694 | FASLG | 178 | 0.001 |
| ENSP00000387006 | CWC22 | 178 | 0.001 |
| ENSP00000332018 | CTAG1A | 1 | 0.001 |
| ENSP00000264474 | CSTA | 178 | 0.001 |
| ENSP00000263168 | CAPZA1 | 178 | 0.001 |
| ENSP00000262584 | RPL8 | 178 | 0.002 |
| ENSP00000355652 | RHOU | 178 | 0.002 |
| ENSP00000327077 | PCM1 | 1 | 0.002 |
| ENSP00000262077 | NUP153 | 353 | 0.002 |
| ENSP00000239223 | DUSP1 | 100 | 0.002 |
| ENSP00000246891 | CSN1S1 | 178 | 0.002 |
| ENSP00000335325 | CCDC73 | 1 | 0.002 |
| ENSP00000242067 | BBS9 | 178 | 0.002 |
| ENSP00000278616 | ATM | 569 | 0.002 |
| ENSP00000368438 | PCNA | 1279 | 0.003 |
| ENSP00000345530 | NEDD4 | 178 | 0.003 |
| ENSP00000415183 | MUC2 | 178 | 0.003 |
| ENSP00000290200 | IL10RB | 178 | 0.003 |
| ENSP00000301838 | FADD | 332 | 0.003 |
| ENSP00000005340 | DVL2 | 351 | 0.003 |
| ENSP00000218388 | TIMP1 | 180 | 0.004 |
| ENSP00000240618 | KLRK1 | 178 | 0.004 |
| ENSP00000268057 | BBS4 | 1 | 0.004 |
| ENSP00000262238 | YY1 | 355 | 0.005 |
| ENSP00000245932 | VASP | 178 | 0.005 |
| ENSP00000263734 | EPAS1 | 17 | 0.005 |
| ENSP00000262768 | TIMP2 | 181 | 0.006 |
| ENSP00000260731 | KIF11 | 178 | 0.007 |
| ENSP00000227752 | IL10RA | 178 | 0.007 |
| ENSP00000412237 | IL10 | 178 | 0.007 |
| ENSP00000410294 | FGFR2 | 178 | 0.007 |
| ENSP00000353701 | DPP3 | 1 | 0.007 |
| ENSP00000242057 | AHR | 493 | 0.007 |
| ENSP00000265428 | WWP1 | 136 | 0.008 |
| ENSP00000369050 | CYP1A1 | 354 | 0.008 |
| ENSP00000351407 | ARNT | 485 | 0.008 |
| ENSP00000265441 | WNT2 | 177 | 0.009 |
| ENSP00000305459 | COG8 | 176 | 0.009 |
| ENSP00000371070 | ATP8A2 | 176 | 0.009 |
| ENSP00000265709 | ANK1 | 178 | 0.009 |
| ENSP00000358866 | FLNA | 178 | 0.01 |
| ENSP00000285018 | WNT7A | 1 | 0.011 |
| ENSP00000290158 | KPNB1 | 351 | 0.011 |
| ENSP00000335620 | GSTA1 | 242 | 0.011 |
| ENSP00000308021 | CEP290 | 178 | 0.011 |
| ENSP00000267082 | ITGB7 | 133 | 0.012 |
| ENSP00000241052 | CAT | 177 | 0.012 |
| ENSP00000337722 | ARL6 | 176 | 0.012 |
| ENSP00000332448 | ADAT3 | 2 | 0.012 |
| ENSP00000294339 | TAL1 | 178 | 0.014 |
| ENSP00000291700 | S100B | 178 | 0.014 |
| ENSP00000259808 | RIPK1 | 326 | 0.014 |
| ENSP00000297338 | RAD21 | 24 | 0.014 |
| ENSP00000364252 | PLA2G2A | 175 | 0.014 |
| ENSP00000257068 | MTNR1B | 6 | 0.014 |
| ENSP00000345344 | CTSL1 | 178 | 0.014 |
| ENSP00000270349 | SLC6A3 | 178 | 0.015 |
| ENSP00000219070 | MMP2 | 228 | 0.015 |
| ENSP00000304592 | FASN | 178 | 0.015 |
| ENSP00000228837 | FGF6 | 178 | 0.016 |
| ENSP00000336868 | CENPA | 178 | 0.017 |
| ENSP00000265517 | MTTP | 5 | 0.018 |
| ENSP00000359211 | DPYD | 177 | 0.018 |
| ENSP00000262965 | TCF3 | 178 | 0.019 |
| ENSP00000276297 | DLC1 | 175 | 0.019 |
| ENSP00000361405 | MMP9 | 186 | 0.02 |
| ENSP00000264634 | WNT5A | 1 | 0.021 |
| ENSP00000408526 | IMPA1 | 39 | 0.021 |
| ENSP00000292853 | FBXO27 | 174 | 0.021 |
| ENSP00000323967 | SMARCE1 | 177 | 0.022 |
| ENSP00000359074 | L1CAM | 178 | 0.022 |
| ENSP00000382133 | DNA2 | 177 | 0.023 |
| ENSP00000371634 | IGF2BP2 | 171 | 0.024 |
| ENSP00000255030 | CRP | 177 | 0.025 |
| ENSP00000312455 | CFLAR | 84 | 0.025 |
| ENSP00000296674 | RPS23 | 77 | 0.027 |
| ENSP00000302021 | MUC7 | 1 | 0.028 |
| ENSP00000211122 | GSTA3 | 1 | 0.028 |
| ENSP00000351997 | MAP2K6 | 141 | 0.029 |
| ENSP00000233146 | MSH2 | 178 | 0.03 |
| ENSP00000314004 | ANAPC2 | 178 | 0.03 |
| ENSP00000361433 | EXOSC2 | 3 | 0.031 |
| ENSP00000262367 | CREBBP | 744 | 0.031 |
| ENSP00000303315 | JUNB | 5 | 0.032 |
| ENSP00000252945 | CYP2E1 | 179 | 0.032 |
| ENSP00000354612 | PTGS1 | 182 | 0.033 |
| ENSP00000376776 | DBH | 173 | 0.033 |
| ENSP00000360025 | GADD45A | 102 | 0.034 |
| ENSP00000245157 | BBS2 | 177 | 0.034 |
| ENSP00000317955 | EEA1 | 3 | 0.035 |
| ENSP00000351486 | NTRK1 | 302 | 0.036 |
| ENSP00000217244 | CSNK2A1 | 178 | 0.036 |
| ENSP00000360266 | JUN | 901 | 0.037 |
| ENSP00000311469 | GSTM1 | 2 | 0.039 |
| ENSP00000346839 | FN1 | 581 | 0.039 |
| ENSP00000379339 | RPS29 | 101 | 0.04 |
| ENSP00000278568 | PAK1 | 182 | 0.04 |
| ENSP00000377865 | RPL23 | 1 | 0.041 |
| ENSP00000305480 | FEN1 | 177 | 0.041 |
| ENSP00000355536 | MTR | 308 | 0.042 |
| ENSP00000258654 | COG3 | 176 | 0.043 |
| ENSP00000359300 | CETN2 | 178 | 0.043 |
| ENSP00000358105 | APH1A | 13 | 0.043 |
| ENSP00000300289 | PDIA3 | 140 | 0.044 |
| ENSP00000382342 | ABCC1 | 169 | 0.044 |
| ENSP00000252029 | TYMP | 6 | 0.045 |
| ENSP00000359939 | EXOSC1 | 1 | 0.045 |
| ENSP00000010338 | TRAF3IP3 | 175 | 0.047 |
| ENSP00000293379 | ITGA5 | 369 | 0.048 |
| ENSP00000364094 | ITGB1 | 418 | 0.049 |
| CID000157350 | hydroxyl radical | 178 | <0.001 |
| CID000010461 | sulfur mustard | 354 | <0.001 |
| CID000031356 | TRIS | 177 | 0.001 |
| CID000004908 | primaquine | 177 | 0.001 |
| CID005324345 | fluvoxamine | 2 | 0.002 |
| CID000440399 | toluene cis-dihydrodiol | 177 | 0.002 |
| CID000082313 | alpha-GlcNAc | 177 | 0.002 |
| CID000002771 | citalopram | 16 | 0.002 |
| CID000002733 | chlorzoxazone | 4 | 0.006 |
| CID000001014 | phosphorylcholine | 178 | 0.006 |
| CID000439655 | d-tartrate | 44 | 0.007 |
| CID000001117 | sulfate | 178 | 0.012 |
| CID000520535 | superoxide | 178 | 0.017 |
| CID000004174 | metyrapone | 7 | 0.017 |
| CID000072886 | L-cysteate | 178 | 0.022 |
| CID000001140 | toluene | 177 | 0.022 |
| CID000119400 | tartrate | 44 | 0.028 |
| CID000000019 | 2,3-dihydroxybenzoic a | 2 | 0.032 |
| CID011020241 | ascorbate | 1 | 0.038 |
| CID000105024 | L-selenomethionine | 311 | 0.039 |
| CID000001775 | phenytoin | 10 | 0.04 |
| CID000003016 | diazepam | 112 | 0.044 |
| CID000030323 | anthracycline | 2 | 0.046 |
| CID000000784 | hydrogen peroxide | 353 | 0.046 |
